# The cumate gene-switch: a system for regulated expression in mammalian cells

**DOI:** 10.1186/1472-6750-6-43

**Published:** 2006-11-03

**Authors:** Alaka Mullick, Yan Xu, René Warren, Maria Koutroumanis, Claire Guilbault, Sophie Broussau, Félix Malenfant, Lucie Bourget, Linda Lamoureux, Rita Lo, Antoine W Caron, Amelie Pilotte, Bernard Massie

**Affiliations:** 1 Institut de Recherche en Biotechnologie, Conseil National de Recherches du Canada, 6100 Royalmount Avenue, Montréal, Québec, H4P 2R2, Canada; 2 INRS-IAF, Université du Québec, Laval, Québec, H7N 4Z3, Canada; 3 Départment de microbiologie et immunologie de l'Université de Montréal, Montréal, Québec, H3C 3J7, Canada; 4 Canada's Michael Smith Genome Sciences Centre, BC Cancer Agency, 570 West 7th Avenue, Vancouver, BC, V5Z 4S6, Canada; 5 Invitrogen, 688 East Main Street, Branford, CT, 06405, USA; 6 AstraZeneca, 7171, Frédérick-Banting, Ville St.-Laurent, Montréal, Québec, H4S 1Z9, Canada

## Abstract

**Background:**

A number of expression systems have been developed where transgene expression can be regulated. They all have specific characteristics making them more suitable for certain applications than for others. Since some applications require the regulation of several genes, there is a need for a variety of independent yet compatible systems.

**Results:**

We have used the regulatory mechanisms of bacterial operons (*cmt *and *cym*) to regulate gene expression in mammalian cells using three different strategies. In the repressor configuration, regulation is mediated by the binding of the repressor (CymR) to the operator site (CuO), placed downstream of a strong constitutive promoter. Addition of cumate, a small molecule, relieves the repression. In the transactivator configuration, a chimaeric transactivator (cTA) protein, formed by the fusion of CymR with the activation domain of VP16, is able to activate transcription when bound to multiple copies of CuO, placed upstream of the CMV minimal promoter. Cumate addition abrogates DNA binding and therefore transactivation by cTA. Finally, an adenoviral library of cTA mutants was screened to identify a reverse cumate activator (rcTA), which activates transcription in the presence rather than the absence of cumate.

**Conclusion:**

We report the generation of a new versatile inducible expression system.

## Background

Tightly controlled inducible expression of transfected genes greatly aids functional studies in relevant biological systems. The ability to regulate both the level and the duration of expression allows the study of proteins whose constitutive expression might not be tolerated by the cell. A number of inducible systems endogenous to mammalian cells involving regulation by heavy-metals [1-3], steroid hormones [4-6], heat shock [7] and other reagents have been developed [8,9]. However, there are limitations with these inducible mammalian promoters such as "leakiness" of the "off" state and pleiotropic effects of inducers (heat shock, heavy metals, glucocorticoids etc.) [1,6]. The use of insect hormones (ecdysone) has been proposed in an attempt to reduce the interference with cellular processes in mammalian cells [10]. Another elegant system uses rapamycin as the inducer [11] but the role of rapamycin as an immunosuppressant was a major limitation to its use *in vivo *and therefore it was necessary to find a biologically inert compound [12] for the control of gene expression.

As an alternative, the control elements from the tetracycline resistance operon from *E. coli *were harnessed for gene regulation in mammalian cells. In its original configuration, an artificial promoter was rendered responsive to a chimaeric transactivator (tTA, composed of the repressor (TetR) fused to the VP16 activation domain), by linking the seven repeats of the Tet operator sequence to the minimal promoter elements of the human cytomegalovirus immediate early gene (CMV-IE) promoter. Transcription from the Tet promoter is activated by tTA whose binding to DNA is inhibited by tetracycline or by the rtTA whose binding to DNA is induced by tetracycline. The specificity of the interaction of TetR with the operator sequence, the large induction levels, the extensive studies on the safety of tetracycline and its high affinity for the TetR made this an attractive system [13]. Since the original report of the Tet switch, a number of modifications have been reported. These include the use of a repressor to block basal transcription [14] and the fusion of a repression domain to the TetR to generate a silencer molecule [15,16]. Furthermore, Hofmann *et al*. [17] have shown that use of a modified mouse mammary tumor virus promoter (MMTV) instead of the minimal CMV promoter in the Tet switch results in a lower basal activity in the absence of transactivator. Baron *et al*. [18] reported that cells better tolerate a modified tTA, wherein the VP16 activation domain is truncated. A first generation reverse mutant was isolated in a bacterial screen and more recently a screen in *Saccharomyces cerevisiae *has led to the identification of rtTA molecules with improved regulatory properties (rtTA2-S2 and rtTA2-M2). These and other modifications have made the Tet switch the most widely used inducible system in mammalian cells [19].

However, there is a need for additional systems for the control of gene expression in mammalian cells as evidenced by several recent reports of new systems [20-23]. It is not only desirable but necessary to have a wide variety of regulatory systems since an increasing number of applications benefit from the possibility of controlling simultaneously but independently, the expression of several genes [9]. Others may require generating combinatorial control systems that would enable, for instance, the functional characterization of gene products involved in cascades [24] such as signal transduction or control of programmed cell death. All of these require the use of several compatible gene regulation systems. Thus, following the paradigm of the TetR, other microbial repressors regulating antibiotic resistance operons, have been developed by Fussenegger and co-workers [25,26]. Both the stretogramin- and macrolide-based gene switches have been shown to be efficient *in vitro *and *in vivo *and fully compatible with the Tet switch. Combination of these gene switches now allows for sophisticated multilevel gene regulation in mammalian cells [27].

**Figure 1:**
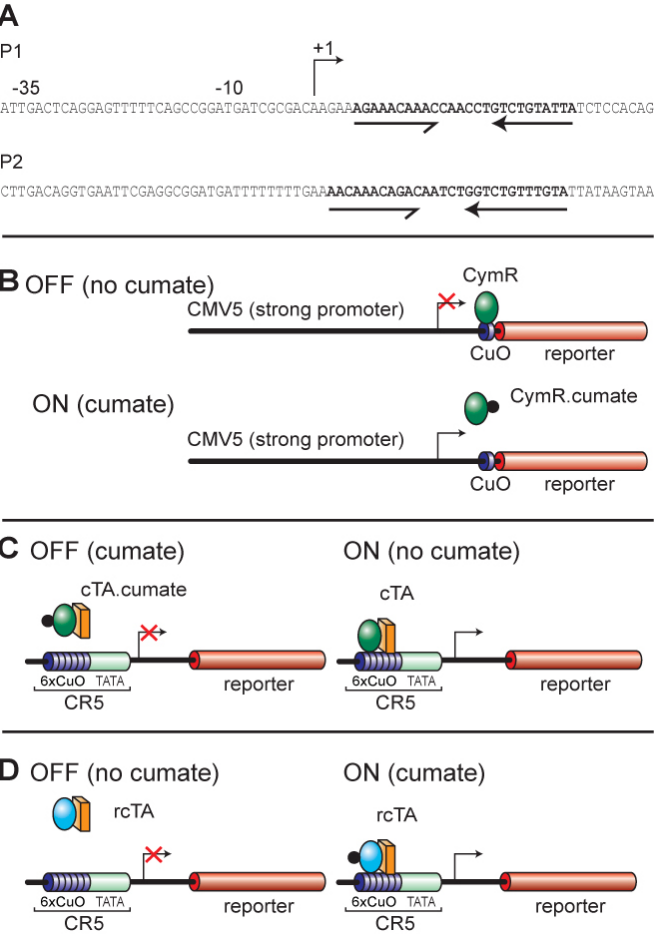
**Schematic representation of the Cumate switch. A. Sequences encompassing the promoter regions of *p-cym *and *p-cmt*: **P1 and P2 are sequences from the promoter region of two bacterial operons regulated by CymR. The arrows represent putative binding sites for a helix-turn-helix CymR repressor. **B. Repressor configuration: **The bacterial repressor, CymR, can bind to the operator sequence (CuO) placed downstream of CMV5, a strong viral promoter that is active in mammalian cells. Once bound, CymR blocks transcription from the CMV5 promoter. CymR bound to cumate is unable to bind to CuO. Transcription from CMV5 can proceed unhindered. **C. Activator configuration: **A chimaeric transactivator, cTA can activate transcription from a minimal CMV promoter by binding to six repeats of the putative DNA recognition sequence (6X-CuO) placed upstream of the promoter in the absence of cumate. Upon cumate addition, the activator no longer binds DNA and therefore is no longer able to activate transcription from the basal promoter. **D. Reverse activator configuration: **A chimaeric transactivator, rcTA can activate transcription from a minimal CMV promoter by binding to six repeats of the putative DNA recognition sequence (6X-CuO) placed upstream of the promoter in the presence of cumate.

The bacterial repressor we have chosen to use as a base for developing our system controls expression from the *p-cmt *and *p-cym *operon in *Pseudomonas putida *[28]. It has a deduced molecular weight of 23,324 daltons. By sequence comparison, it has been proposed that the DNA-binding domain is located in the N-terminus of the protein and has the characteristics of a helix-turn-helix motif. DNA sequence analysis of the promoter regions (P1 and P2) (Fig. 1A) of the *p-cym *and *p-cmt *operons, reveal an imperfect and a perfect inverted repeat (in bold; Fig 1A) respectively with characteristics of a binding site for a helix turn helix DNA-binding domain [28]. The imperfect repeat is located between the promoter and the beginning of the first gene in the *p*-cymene degradative pathway. This gene codes for cumic alcohol dehydrogenase, which is responsible for the conversion of *p*-cymene to *p*-cumate. A similar sequence is found in the promoter region of the Da gene, which is first in the degradative pathway of *p*-cumate. Since CymR regulates expression from the *p*-*cym *and *p*-*cmt *operons, both sequences have been defined as putative operator sequences (CuO).

We have exploited CymR and CuO to control gene expression in mammalian cells using three different strategies (Fig. 1).

1) Repressor configuration: CymR was used to repress transcription from a mammalian promoter by binding an operator site (CuO) placed downstream of the initiation site. Addition of the inducer (cumate) to mammalian cells causes a change in the configuration of CymR such that it can no longer bind DNA and thus relieves repression (Fig 1B).

2) Activator configuration: CymR was fused to an activation domain and the chimaeric molecule (cTA) was used to activate transcription from a minimal promoter downstream of multimerized operator binding sites (6X-CuO). Again binding of cTA, and therefore activation, is regulated by addition of cumate (Fig. 1C).

3) Reverse activator configuration: rCymR was fused to VP16 to give rise to the chimaeric molecule (rcTA) which was used to activate transcription from a minimal promoter downstream of multimerized operator binding sites (6X-CuO). Binding of rcTA, and therefore activation, is induced by addition of cumate (Fig. 1D).

In this report we describe the generation and testing of the three different configurations of the switch in transient and stable transfectants.

## Results

### The repressor configuration

#### Transient transfections

**Figure 2:**
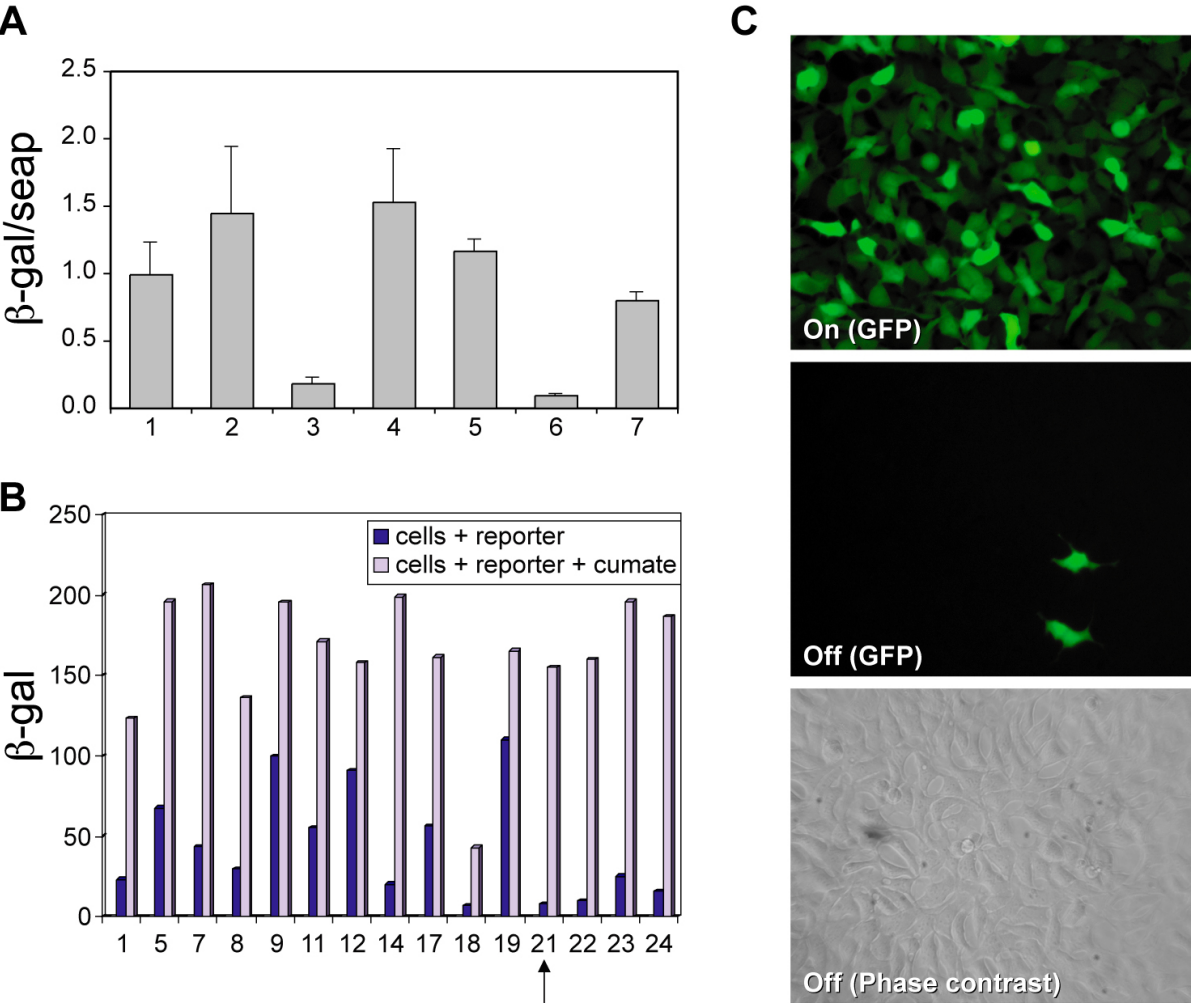
**The Repressor configuration. A. Transient transfection: **Five micrograms of reporter (pAdCMV5-CuOs-LacZ or pAdCMV5-CuOg-LacZ) were transfected alone (lanes 2 and 5 respectively) or co-transfected with 250 ng repressor construct (pAdCMV5-CymR) (lanes 3, 4 and 6, 7) in 293 cells. Three micrograms of pcDNA-SEAP was included as an internal control of transfection efficiency. Transfections were carried out in the presence (4, 7) and absence (lanes 1, 2, 3, 5 and 6) of 200 μg/ml cumate. β-galactosidase (β-gal) activity was measured 48 h post-transfection using a colorimetric assay. Reporter activity was normalized to SEAP activity in the culture medium. pAdCMV5-LacZ was used as a reference for promoter strength (lane l). The figure represents data from 3 independent experiments. **B. Screening 293-CymR clones: **Clones of 293 stably expressing CymR were infected with AdCMV5-CuOs-LacZ (MOI 10) in the presence and absence of 200 μg/ml cumate. β-galactosidase activity was measured 48 h post-infection using a colorimetric assay in cell extracts. Clone 21, marked with an arrow was retained for further experimentation. **C. Stable expression of reporter: **293-CymR-CMV5-CuO-GFP cells were cultured in the presence and absence of 200 μg/ml of cumate for 48 h. The figure shows flurescent and phase contrast images.

To assess the potential of the regulatory elements of the *p-cymene *operon for regulation of gene expression in mammalian cells, we first examined the ability of CymR to down-regulate the expression of a strong viral promoter (CMV5: CMV-promoter-enhancer sequences modified by the addition the Adenoviral tripartite leader sequence and downstream enhancer elements in the intron 3' to the transcription initiation site) in mammalian cells (Fig. 1B). The CymR coding sequence was therefore cloned in an expression vector (pAdCMV5-K7-GFP) downstream of the CMV5 promoter to generate pAdCMV5-CymR. 293 cells were co-transfected with pAdCMV5-CymR, and with, either pAdCMV5-CuOs-LacZ wherein, expression from CMV5 can be blocked by the binding of CymR to CuO inserted at the AscI site at the start site of transcription (CuOs) or pAdCMV5-CuOg-LacZ, wherein CuO is inserted 10 bases downstream of the TATA box (CuOg). Fig. 2A shows that when the reporter construct is transfected alone, high-level expression is detected (lane 2). It is important to note that these levels are comparable to those from a parental construct, pAdCMV5-LacZ, lacking the operator site (lane 1). On co-transfection with the plasmid pAdCMV5-CymR, 95% of the activity is blocked by CymR expression (lane 3). In addition, repression is totally relieved upon the addition of cumate (lane 4). Similar results are seen when the operator site is introduced in the Age I site in the reporter construct (pAdCMV5-CuOg-LacZ) (lanes 5–7). In all subsequent DNA constructs, the configuration corresponding to pAdCMV5-CuOs-LacZ was used and the position is no longer specified with a suffix.

#### Stable expression in 293 cells

Having observed tight control in transient transfections, we determined whether expression could be similarly regulated in cell lines stably expressing CymR. Therefore the CymR coding sequence was cloned into an expression plasmid, pMPG-tk-neo, containing an expression cassette for the neomycin resistance gene. 293 cells were transfected with pMPG-CymR/tk-neo and a pool of G418-resistant cells were isolated. To screen for clones that expressed levels of CymR optimal for tight regulation, they were infected with AdCMV5-CuOs-LacZ reporter AdV (an AdV expressing β-galactosidase under the control of the CMV5-CuO promoter) and LacZ expression was measured in the absence and presence of cumate (Fig 2B). The degree of repression varied between 1.7 and 19.4 fold. In general, clones that showed a high degree of repression were more difficult to activate and *vice versa*. 293CymR-clone 21 was identified as one that exhibits a combination of efficient repression (ON/OFF ratio 19) and activation. It was thus chosen for the next step, which was to express the reporter construct stably.

A reporter plasmid, pAdCMV5-CuO-GFP, containing a GFP expression cassette wherein GFP expression is regulated by the CMV5-CuO promoter, was stably expressed in 293CymR clone 21 to generate 293CymR-CMV5-CuO-GFP clones. Figure 2C shows the performance of the switch in such a configuration using one of the clones generated. In the ON state, most cells displayed strong GFP fluorescence, whereas in the OFF state, only two cells were visible with fluorescence microscopy although the phase contrast image shows a dense cellular monolayer. The fluorescence index (percentage of GFP positive X mean fluorescent signal) was measured in the ON (9255) and OFF (37.5) states by flow cytometry. The ON/OFF ratio for this clone was 246.

### The activator configuration

#### Transient transfections

**Figure 3:**
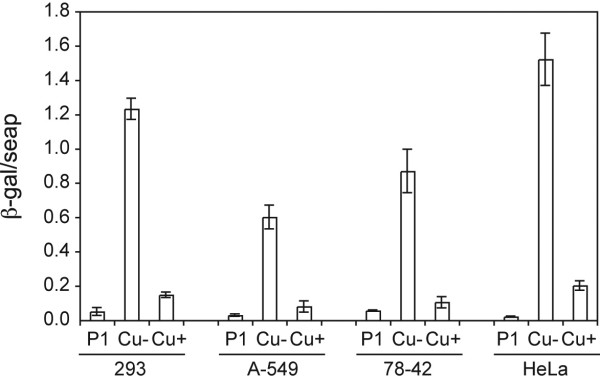
**Activator configuration: Transient transfection: **Reporter construct (pAdCR5-LacZ) was transfected alone (P1) or was co-transfected with activator construct, pAdCMV5-cTA (Cu- and Cu+), in 293, A549, BMAdE1-78 and HeLa cells. pcDNA-SEAP was included to serve as a control for transfection efficiency. Transfections were carried out in the presence (Cu+) and absence (Cu-) of 200 μg/ml cumate. β-galactosidase activity was measured 48 h post-transfection. Reporter activity was normalized to SEAP activity in the culture medium. The figure represents data from 3 independent experiments.

To transform CymR into a transactivator, it was fused to the VP16 activation domain such that this chimaeric molecule could activate transcription by binding multimerized CuO elements (6X-CuO) upstream of a minimal promoter (Fig. 1C). To this end, the sequences coding for this chimaeric molecule (cTA) were cloned into an expression vector, pAdCMV5-K7-BFP giving rise to pAdCMV5-cTA, where expression of cTA is controlled by the CMV5 promoter. To test the system, pAdCMV5-cTA was co-transfected with a reporter, pAdCR5-LacZ wherein six repeats of the operator sequence are placed upstream of the CMV minimal promoter. Reporter and activator constructs were co-transfected into either 293, HeLa, A549 or BMAdE1 (a derivative of A549 stably expressing the adenovirus E1 region) cells. Fig. 3A shows that the reporter construct, pAdCR5-LacZ, when transfected alone, produced minimal amounts of β-gal activity. On co-transfection with the plasmid coding for the transactivator, there was a large increase in the β-gal activity. Addition of cumate to the medium reduced the activation to different extents in the various cell lines, resulting in ON/OFF ratios of 8.6 +/- 2.1, 13 +/- 7.8, 10.3 +/- 6.0 and 7.7 +/- 0.95 in 293, A549, BMAdE1-78, and HeLa cells respectively. Both operator sequences (P1 and P 2; Fig. 1A) perform equally well to mediate transactivation (data not shown), therefore all subsequent experiments were performed with the multimerized P2 elements linked to the CMV TATA box (CR5 promoter).

#### Stable expression in 293 cells

The cTA hybrid gene was cloned into an expression plasmid, pcDNA, containing an expression cassette for the neomycin resistance gene. 293 cells were transfected with pcDNA-cTA and a pool of G418-resistant cells were isolated. Less than 10 clones were obtained, which were screened by infection with AdCR5-LacZ reporter AdV (an AdV expressing β-galactosidase under the control of the CR5 promoter). Clone 30 was chosen for the highest level of activity and the best ON/OFF value, and further sub-cloned by limiting dilution. Reporter gene activity in 5 of the sub-clones were close, varying between 4.9 and 7.8. Clone 30–37 has the best ON/OFF ratio (7.8) and was thus chosen for further experimentation. It is noteworthy that, following adenoviral infection in 293 cells, the expression cassette is amplified to greater than 10E5 copies per cell and, as a result, the basal level of expression from the minimal promoter is fairly high as reported earlier [29]. Therefore the efficacy of the cTA to affect tight regulation from the CR5 promoter was further examined under low copy number as is the case in stable expression systems. Thus the selected 293cTA clone (clone 30–37) was transduced with reporter lentivirus, Lenti-CR5-GFP, wherein GFP expression is regulated by the CR5 promoter, giving rise to 293cTA-CR5-GFP pool. Reporter gene expression of the 293cTA-CR5-GFP was studied in comparison to 293CymR-CMV5CuO-GFP for dose response to cumate as follows.

### Dose response of the repressor and activator configurations

**Figure 4:**
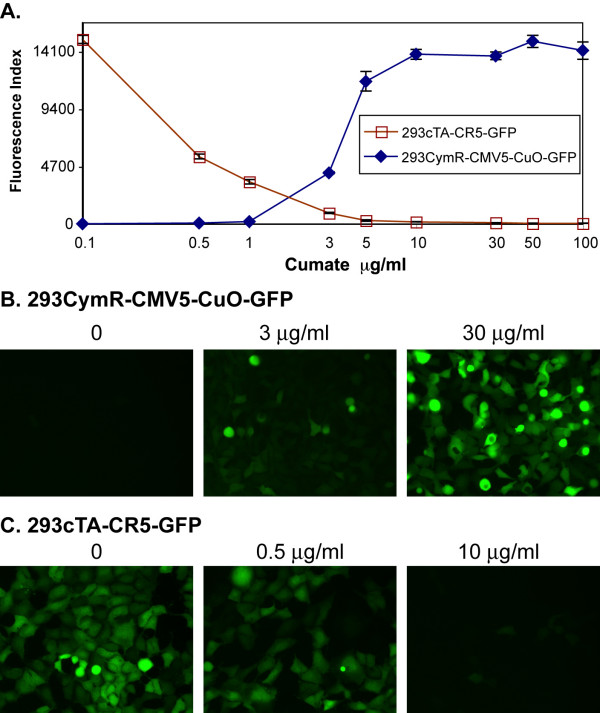
**A. Dose-dependent control of reporter gene expression by cumate: **293CymR-CMV5-CuO-GFP and 293cTA-CR5-GFP cells that were cultured under conditions where reporter gene expression was off (293CymR-CMV5-CuO-GFP in the absence of cumate and 293cTA-CR5-GFP, in the presence of 50 μg/ml cumate) were washed with PBS and incubated for 48 h in the presence of varying concentrations of cumate. Total GFP fluorescence was measured by flow cytometry and is indicated in the y-axis in terms of fluorescence index (% GFP positive cells X mean fluorescence index). The figure represents the data from triplicate measurements. **B. Tight control of GFP expression in cells stably expressing CymR and pCMV5-CuO-GFP: **A clone of 293CymR-CMV5-CuO-GFP was cultured for 48 h in the presence of various concentrations of cumate. The figure shows representative fluorescent images of the cells treated with 0, 3 and 30 μg/ml of cumate from the experiment shown in the panel A. **C. Tight control of GFP expression in cells stably expressing cTA and pCR5-GFP: **The enriched pool of 293cTA-CR5-GFP, that was cultured in the presence of cumate, was washed in PBS and incubated for 48 h in the presence of various concentrations of cumate. The figure shows representative fluorescent images of the cells treated with 0, 0.5 and 10 μg/ml of cumate from the experiment shown in the panel A.

To determine whether the control of gene expression was dose-dependent, cell lines stably expressing the repressor (293CymR-CMV5-CuO-GFP) or activator (293cTA-CR5-GFP) configurations of the switch were cultured under conditions where gene expression is OFF (293CymR-CMV5-CuO-GFP in the absence of cumate and 293cTA-CR5-GFP in the presence of cumate). It is important to keep gene expression OFF while isolating a cell line stably expressing a gene of interest, because the expression of certain proteins may interfere with cell growth and thus with the process of isolating stable clones. To initiate the experiment, cells were washed and incubated in different concentrations of cumate. GFP expression was measured by flow cytometry, 48 h later. In the case of 293CymR-CMV5-CuO-GFP GFP fluorescence was almost at background levels (% GFP+ cells = 1.45 and mean fluorescence intensity (MFI) = 6, Fluorescence index = 8.7) without the addition of cumate (Fig. 4). Upon the addition of 3 μg/ml of cumate 93% of the cells are GFP +, with a MFI of 9.5, giving a fluorescence index of 883. Further addition of cumate increases the MFI to 25.2 and 29.3 at 5 μg/ml and 10 μg/ml cumate respectively. At 10 μg/ml, the switch is maximally activated, since further addition of cumate (up to 100 μg/ml) does not result in any significant increases in MFI (30). The maximal ON/OFF ratio is thus 330.

In the 293cTA-CR5-GFP cell pool generated by lentiviral transduction of the reporter, the switch is maximally ON, 65 % cells being GFP+ with an MFI of 49, without the addition of cumate (Fig. 4(i) and (iii)). Upon the addition of 0.5, 1.0, 3.0, 5.0, 10.0 30.0, 50.0 and 100.0 μg/ml of cumate 46%, 38%, 19%, 9%, 6%, 5%, 3% and 2% of the cells are GFP +, with MFIs of 25, 19, 10, 7, 6, 5, 6 and 6 respectively. At 30 μg/ml, the switch is completely OFF, since additional increases in the cumate concentration does not cause a significant reduction in, either the percentage of GFP+ cells or the MFI. The maximal ON/OFF ratio is thus 237.

### Reverse activator configuration

To further increase the versatility of the cumate switch, we undertook a screen of CymR mutants to isolate a variant that would bind DNA in the presence rather than the absence of cumate. We chose to perform the screen in mammalian cells using an adenoviral vector for delivery of the mutant library [30,31].

#### Screening an adenoviral library of CymR mutants

##### Generating host cell line: 293CR5-LacZ

**Figure 5:**
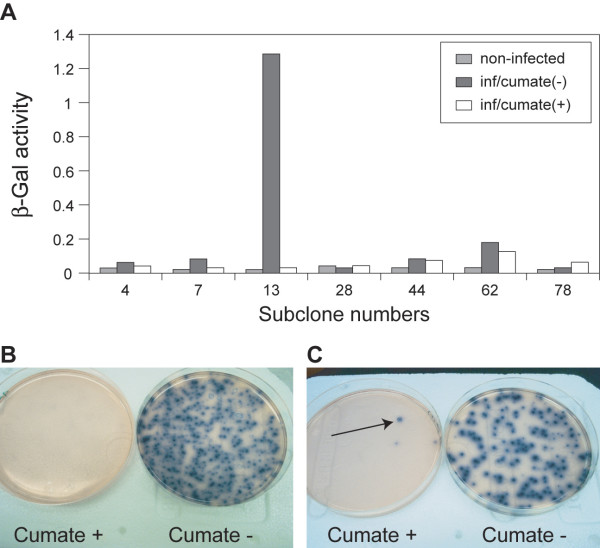
**A. Screening of 293-CR5-LacZ clones**. G418-resistant clones were infected with AdCMV-cTA in the presence and absence of 200 μg/ml cumate. Forty-eight hours later, β-Gal activity was measured. The figure shows the subclone # 13 displays high-level expression and the best ON/OFF ratio, almost 40-fold. **B. Screening adenoviral library of cTA mutants. **293CR5-LacZ cells were infected with the adenoviral library of CymR mutants and overlayed with agarose supplemented with Blue-gal. Plaques formed by the infection of adenovirus expressing the wild-type cTA are β-gal positive in the absence of cumate, and β-gal negative in the presence of 200 μg/ml cumate. **C. **The plaque indicated by an arrow is a candidate for the reverse phenotype, since high β-gal expression is evident in the presence of 200 μg/ml cumate.

In order to efficiently screen the library of the mutated transactivator, cTA, a stable 293 cell line with optimized sensitivity for cumate induction was established by stable expression of a β-galactosidase reporter, pAdCR5-LacZ-neo in 293 cells. Fig. 5A shows the reporter activity of 7 of the resulting G418-resistant clones when infected with an AdV expressing the transactivator (AdCMV5-cTA) in the presence and absence of cumate. Of the more than 100 clones screened, Clone 13 was retained for further analysis since it displayed both a strong level of induction and the best ON/OFF ratio (40X). As shown in Fig. 5B, when AdCMVcTA was plated on 293CR5-LacZ at 100–300 plaques per plate, the plates are clearly white in the presence of cumate and blue in the absence of cumate as a result of β-galactosidase expression.

#### Screening the adenoviral library

Error prone PCR was used to generate a library of mutated CymR molecules. Three PCR products with varying levels of nucleotide mis-incorporation, ranging from 0–3 to 7–16 mutations/kb were obtained and subcloned into plasmid pAdPSCMV-cTA-DC-GFP, where they replaced the wild-type CymR. Thus three different libraries with varying frequencies of mutations, each with approximately 2.5 × 10^5 ^recombinant *E. coli *clones, were established. The plasmid pools were used as transfer vectors to generate adenoviral libraries using the positive selection system [30].

The library was plated on 293-CR5-LacZ cells in the presence and absence of cumate, to screen for plaques that were β-galactosidase positive in the presence of cumate as opposed to the wild-type cTA where plaques are β-galactosidase positive in the absence of cumate. Approximately 60,000 plaques were screened. However, only 15 gave the desired phenotype and were therefore picked for analysis. Of these, the majority were not regulated by cumate. Only one mutant satisfied the criteria for being a reverse mutant.

#### The reverse activator in transient transfections

The mutated CymR sequence was amplified from the viral DNA and cloned into pAdCMV5cTA replacing the wild type CymR to give rise to pAdCMV5rcTA. Sequence comparison of the wild-type and mutated DNA revealed three mutations (Ala^125^→Val^125^, Glu^142^→Gly^142 ^and Met^144^→Ile^144^) in the 3'-half of the cDNA.

**Figure 6:**
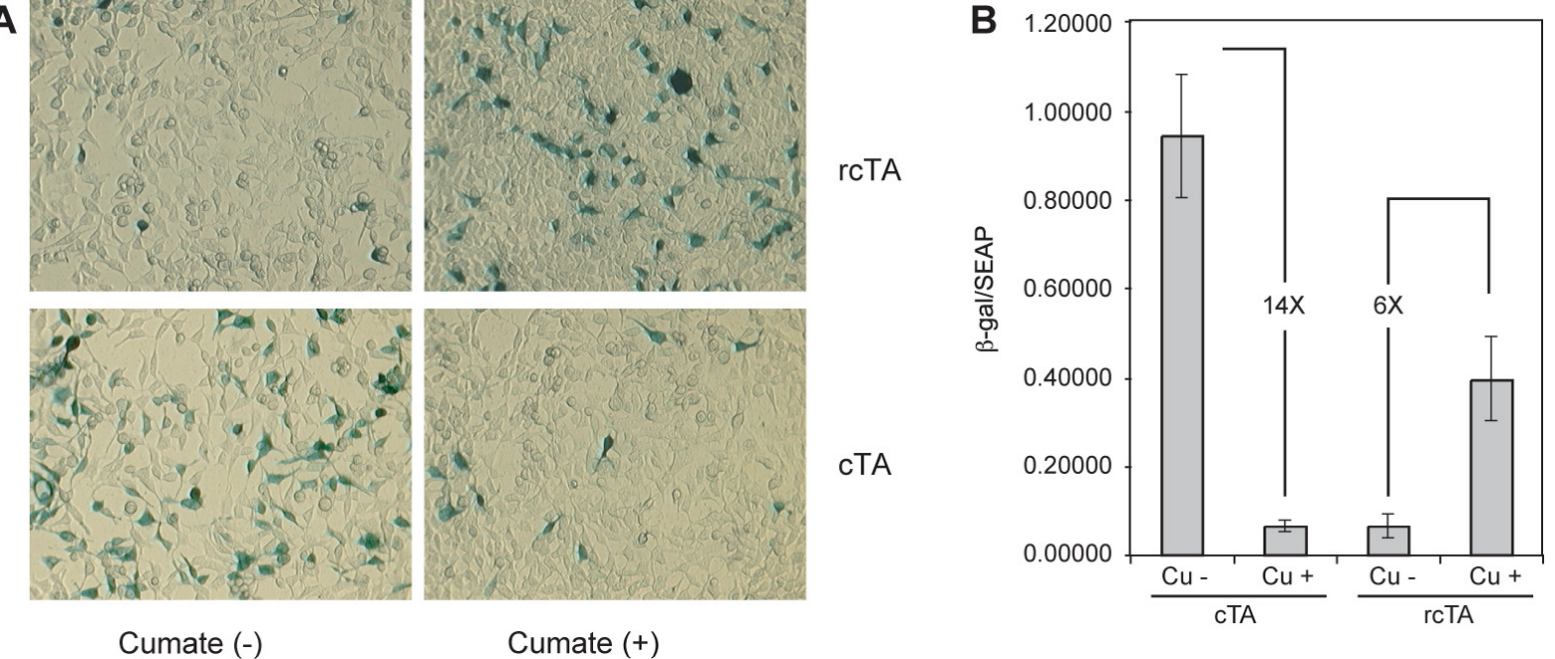
**Comparison of cTA and rcTA activity and expression: A. **293-CR5-LacZ cells were transfected with pAdCMV5-cTA or pAdCMV5-rcTA in the absence or presence of 200 μg/ml cumate. Forty-eight hours later the cells were stained for β-gal activity. **B. **293-CR5-LacZ cells were co-transfected with pcDNA-SEAP and pAdCMV5cTA or pAdCMV5-rcTA in the presence or absence of 200 μg/ml cumate. β-galactosidase activity was measured 48 h post-transfection. Reporter activity was normalized to SEAP activity in the culture medium. The figure represents data from 3 independent experiments.

Transient transfections were performed to further characterize the reverse phenotype of the mutated CymR Fig. 6). As seen in Fig. 6A, whereas β-galactosidase activity is induced in the absence of cumate in the case of the cTA, it is induced in the presence of cumate, in the case of the rcTA. Quantitation of the transfection results are shown in Fig. 6B. The rcTA activates transcription in the presence of cumate, with an ON/OFF ratio of 6. The cTA, on the other hand, regulates transcription more efficiently, with an ON/OFF ratio of 14, despite the fact that it can activate to higher levels (Fig. 6B).

#### Regulation of expression of the reverse activator

**Figure 7:**
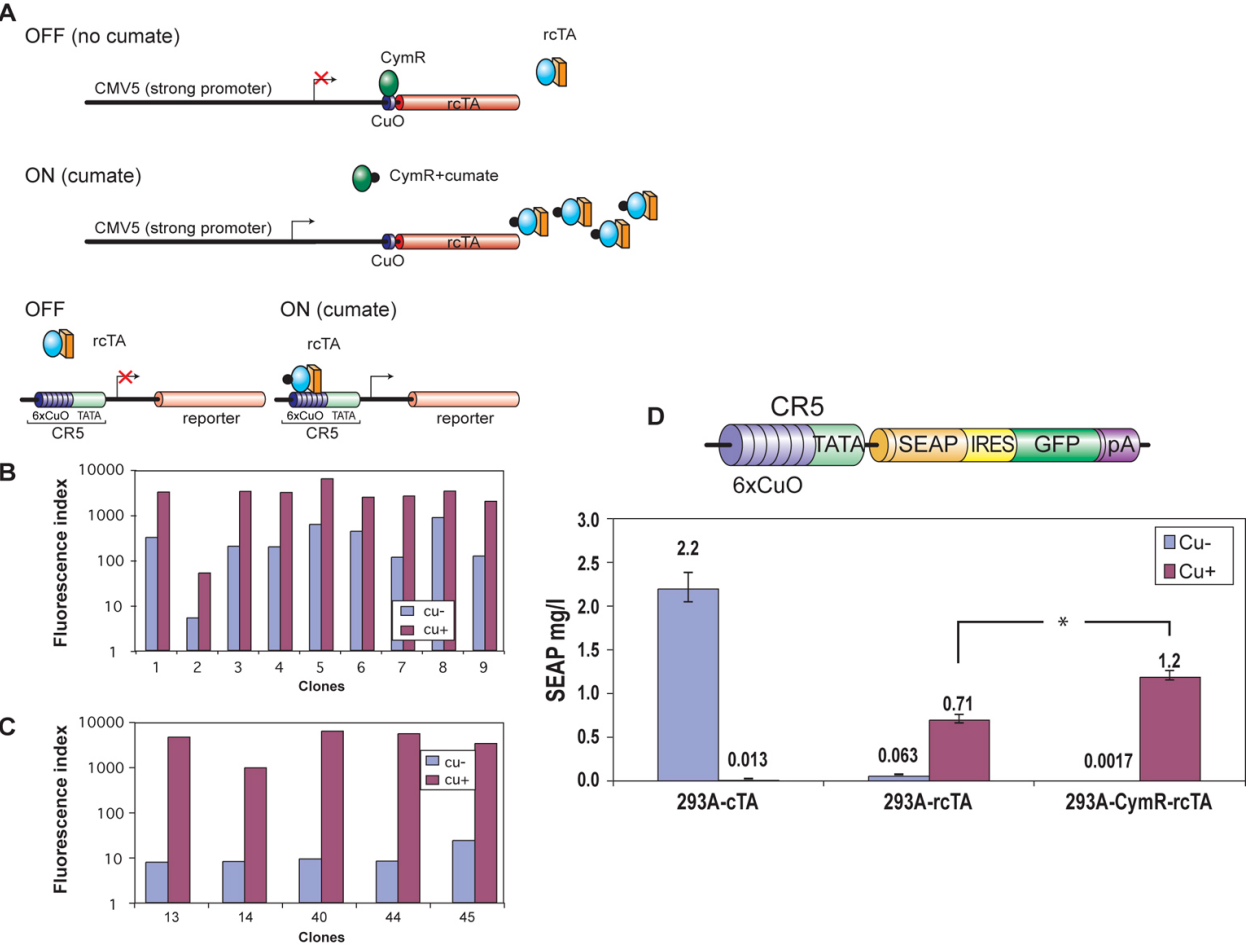
**Regulating rcTA expression. A. Schematic representation of the strategy. **The expression of rcTA is controlled by the CMV5-CuO promoter. In the absence of cumate, CymR binding to CuO blocks the synthesis of rcTA. In the absence of rcTA, GFP expression from CR5-GFP is not stimulated. Leaky expression of rcTA is not sufficient to activate transcription from the CR5 promoter. Upon addition of 50 μg/ml cumate, CymR binding to CuO is abrogated and therefore rcTA is synthesized. Cumate binding to rcTA activates its DNA binding activity thereby turning on reporter gene expression. **B. Screening of 293-rcTA **and **C. 293CymR-rcTA clones. **The clones were infected with lenti-CR5-GFP in the presence and absence of 50 μg/ml cumate and 48 h later GFP fluorescence was measured by flow cytometry. **D. Stable expression of reporter CR5-SEAP-IRES-GFP in 293-rcTA and 293CymR-rcTA: **293cTA, 293-rcTA and 293CymR-rcTA transduced with lenti-CR5-SEAP-IRES-GFP were cultured in the presence and absence of 50 μg/ml cumate for 48 h. SEAP activity was measured in the cell culture medium.

Although the mutant has the desired phenotype, we wanted to explore the possibility of improving its ability to regulate gene expression. We thus regulated the expression of the rcTA using a strategy that is illustrated in Fig. 7A. We hypothesized that in the absence of cumate, CymR would block the expression of rcTA from the plasmid pAdCMV5-CuO-rcTA, maintaining reporter gene expression from the rcTA-dependent pAdCR5-LacZ, at minimal levels. In the presence of cumate, however, both the expression and the activation function of the rcTA should be induced and reporter gene expression will be at maximal levels. To experimentally verify the advantage of this strategy, two cell lines stably expressing rcTA were generated. A lentiviral vector expressing the rcTA under the control of the CMV5-CuO promoter was used to transduce the parental 293 cells and a clone of 293 (293CymR) that stably expresses CymR, thus regulating expression from the CMV5 promoter (described above in section on the repressor configuration). The two cell pools (293-rcTA and 293CymR-rcTA) were sub-cloned by limiting dilution. The level of reporter gene expression upon lentiviral infection of representative 293rcTA and 293CymR-rcTA clones with lentiCR5-GFP is shown in Fig. 7B and 7C respectively. The superior level of regulation in the 293CymR-rcTA sub-clones is evident. To better assess the induction factor, SEAP was subsequently used as a reporter since it allows for more precise measurements than GFP in cases of low-level expression, such as un-induced conditions. 293-CymR-rcTA#40 and 293rcTA#18 were retained for transduction with a lentiviral reporter (lenti-CR5-SEAP-IRES-GFP). For the sake of comparison, 293 cells stably expressing the cTA (293cTA described in section on the activator configuraion) were also transduced with the same reporter lentivirus. By monitoring GFP expression, the three pools were deemed to contain a similar percentage of transduced cells (76, 52 and 72 for 293cTA-CR5-GFP, 293rcTA-CR5-GFP and 293CymR-rcTA-CR5-GFP respectively). The results for the three cell pools are shown in Fig. 7D, which demonstrated a significant increase in the ON/OFF ratio by regulating the expression of the rcTA. The data also shows that the double regulation result in superior induction factor (700-fold) as compared to the 293-rcTA due to significant reduction in basal level under un-induced condition.

## Discussion

We have described the construction of a new inducible system for expression in mammalian cells. We have adapted the regulatory mechanism of a bacterial operon derived from *Pseudomonas putida *to a mammalian expression system using three different strategies (Fig. 1). Since cumate is the effector molecule that regulates the CymR-mediated expression and therefore CymR-DNA binding, it is the molecule we have used to regulate expression from the mammalian expression system incorporating CymR. It is important to note that, at concentrations that effectively control gene expression, mammalian cell growth is not affected over 10 passages (data not shown).

The different strategies we have chosen to build an inducible system for expression in mammalian cells have been used before and have proven to be highly successful [9,19,32]. This approach is particularly attractive because it lends itself very well to improvement as a consequence of its modular nature. A very well studied example of a chimaeric transactivator is the tTA where the Tet repressor has been transformed into an activator by fusion with the VP16 activation domain [13]. The repressor is fused to an activation domain, the two modules being functionally independent. It is possible thus to improve and exchange the activation domain without affecting repressor function. Modifications in the VP16 transactivation domain have been identified that render it less toxic, while maintaining its activation potential [18]. It is similarly possible to modify the DNA-binding or dimerization properties of the repressor and leave the transactivation function unchanged [33,34]. A large number of such improvements have been described for the Tet system in the literature (reviewed in ([9,19,35]). More recently, macrolide- and streptogramin-based gene-switches were also modified so as to render them more versatile [26].

In the first strategy CymR is used as a repressor that reversibly blocks expression from a strong promoter. The operator sequence was inserted at two different sites with respect to the TATA box since there is some debate in the literature as to the role of site of insertion. A detailed study by Hu and Davidson wherein lac operator sequences are inserted at different positions in the SV40 promoter region, indicates that in all cases there is a decrease in promoter activity due to the insertion *per se *[36]. More recently, Hedengren-Olcott and Hruby [37] have demonstrated that the activity of the vaccinia virus *GIL *promoter is reduced by the insertion of the *tetO *sequence. In our case, insertion in two different positions (between the TATA box and the initiation site or just downstream of the initiation site) did not affect expression (Fig. 2A). Similarly, Yao *et al*. [14] did not see any decrease in promoter activity as a result of the insertion of the Tet operator site. They reported efficient repression and attributed the success of their strategy to the positioning of the operator site. The positioning was such that the operator was 10 base pairs downstream of the TATA box, resulting in repressor binding on the same side of the helix as the RNA polymerase. The repressor is therefore able to sterically block the polymerase most effectively. In our case it is not possible to make a definitive prediction regarding DNA-CymR interaction, since the exact sequence requirements for CymR binding to DNA are not yet known. Work is in progress to define sequences, both in the repressor and in the DNA, that participate in this interaction.

Tight control of transgene expression in a cell line stably expressing CymR is evident by examining GFP expression in 293-CMV5-CuO-GFP cells. Fig. 4B allows a visual appreciation of the tight control in the OFF state. Such tight regulation of gene expression is comparable to that reported for established cell lines being used in inducible expression systems [14,38,39]. Although this strategy offers great promise for stable expression of a heterologous protein, it is limited by the strength of the CMV5 promoter in the relevant cell line.

To generate a regulatory system that would be more versatile in its application, we also generated a configuration of the switch that would be relatively independent of cellular transcription factors. The chimaeric transactivator, cTA, which is active in several cell lines tested (293, A549, BMAdE1 and HeLa (Fig. 3A) and Peer, CHO (data not shown) can activate transcription from multimers of either the perfect or imperfect repeat. The perfect palindrome, may be expected to be a better binding site for CymR. However, in our assay no significant difference was observed between the two sequences (data not shown). Perhaps, the differences in the two halves of the imperfect repeat are not in critical bases. Moreover, in both cases six copies of the putative recognition sequence are used. Cooperative binding of several activator molecules to the multimerized site may overcome any minor difference in binding activity to individual sites.

Depending upon the application in question, it may be necessary to partially turn the expression of a gene of interest, on or off. We therefore investigated the dose response of reporter gene expression in both repressor and activator configurations of the switch. We chose to use flow cytometry since it has the advantage of providing information both on the fraction of cells expressing transgene and the intensity of transgene expression, as opposed to techniques where global expression is measured in a cellular extract (for example, luciferase). Our data reveal changes at both levels, fraction of cells expressing transgene and the intensity of transgene expression upon the addition of cumate and show that, indeed reporter gene expression is regulated in a dose-dependent manner.

As expected, in the activator configuration, transgene expression is ON in the absence of cumate, which for certain applications would require culturing cells in the presence of cumate to keep transgene expression off until appropriate. Moreover, cumate would have to be removed from the culture medium to turn expression ON. This can be problematic for different reasons including the delay in turning on expression related to the incomplete removal of cumate and the inconvenience of removing cumate from very large culture. We thus carried out a screen of mutant transactivators to identify a reverse mutant. A similar screen was carried out to identify the reverse Tet activator, rtTA. A first screen in a bacterial system identified the first generation of rtTA [33]. However, a reverse mutant with better regulatory properties was isolated in a later screen in a eukaryotic cell type, *Saccharomyces cerevisae *[40]. To increase the probability of identifying a mutant with optimal function in mammalian cells, we undertook the screen in 293 cells. However, this is a task with several technical challenges, a major one being the isolation of a host cell that has taken up the desired mutant and presuming this is successful, then, the isolation of the mutated DNA from the cell. We thus used adenoviral vectors to deliver the library, since plaque-formation results in the localized amplification of individual AdV and isolation of the mutated DNA from the adenoviral genome is straightforward. Our proprietary technology for the positive selection of recombinant adenoviruses further facilitated the task [30]. Of all the mutants screened, only one had the desired reverse phenotype with respect to regulation of CuO binding by cumate. The rcTA thus isolated, although functional, is sub-optimal in its regulatory function. All three mutations are in the 3'-half of the cDNA. Work is in progress to gain an insight into the structure-function analysis of the CymR to rationally design mutants with optimized cumate-regulated DNA binding properties. In the mean time we decided to take advantage of the opposite regulation by cumate of DNA binding of CymR and rcTA similar to what was done for the Tet system by Freundlieb *et al*.[16]. We thus controlled the expression of the rcTA using CymR and generated cell lines with optimal regulation of gene expression in the reverse configuration and were thus able to obtain tight control of gene expression which is relatively cell-type independent and can be turned ON with the addition of the inducer molecule, cumate.

## Conclusion

In summary, we have demonstrated the feasibility of harnessing the regulatory elements of the *p-cym *operon from *Pseudomonas putida *to regulate gene expression in mammalian cells. In both the repressor and activator configurations, we have demonstrated tight control of gene expression both in transient and stable expression. Although both systems have advantages, as might be expected, they also have limitations for various applications. To use the repressor configuration, it is imperative that a suitable promoter be available for the cell line in question. Although this is rectified with the use of the cTA, gene expression cannot be turned ON by the addition of an inducer in this case. There is thus a need for the removal of cumate, which could be inconvenient in situations such as large-scale protein production. To increase the versatility of the expression system, we have isolated a reverse mutant, which allows gene expression to be turned on in the presence rather than absence of cumate. Although the reverse mutant is sub-optimal, we have combined two configurations of the switch to generate tight control of gene expression. We thus have a versatile system with various configurations.

## Methods

### Plasmids

All plasmids named with pAd are adenoviral transfer vectors containing viral sequences between 0 – 1.4 and 9.4 – 15.5 map units that are required for homologous recombination to generate Ad recombinants. Their presence should not result in the synthesis of viral proteins. The plasmids used in this study are listed in Table 1.

**Table 1:** List of plasmids used in this study.

**Plasmid name**	**Configuration**	**Promoter**	**Coding sequence**	**Purpose**
**pAdCMV5-CuOg-LacZ**	Repressor	CMV5-CuO	LacZ	Reporter for transient transfection
**pAdCMV5-CuOs-LacZ**	Repressor	CMV5-CuO	LacZ	Reporter for transient transfection and adenovirus for screening stable transfectants of CymR
**pAdCMV5-CuO-GFP**	Repressor	CMV5-CuO	GFP	Reporter for stable expression of repressor configuration
**pAdCMV5-CymR-K7-BFP**	Repressor	CMV5	CymR	Repressor expression in transient transfection assays
**pMPG-BFP/CMV5- CymR/tk-neo**	Repressor	CMV5	CymR	Stable expression of CymR
**pAdCR5-LacZ**	Activator	CR5	LacZ	Reporter gene for transient transfection.
**pAdCMV5-cTA- K7BFP**	Activator	CMV5	cTA	Expression of activator for transient transfection
**pcDNA-cTA**	Activator	CMV	cTA	Stable expression of activator
**pAdPS-CMV-cTA-DC- GFP**	Activator	CMV	cTA	Transfer vector for generating adenoviral library for mutant cTAs
**pAdCMV5-rcTA- K7BFP**	Reverse activator	CMV5	rcTA	Expression of reverse activator for transient transfection
**pRRL.cppt.CMV5- CuO-rcTA.WPRE**	Reverse activator	CMV5-CuO	rcTA	Vector for generating lentivirus expressing the rcTA
**pRRL.cppt.CR5- GFP.WPRE**	Activator/Reverse activator	CR5	GFP	Reporter for lentiviral transduction
**pRRL.cppt.CR5-SEAP-IRES-GFP.WPRE**	Activator/Reverse activator	CR5	SEAP and GFP	Reporter for lentiviral transduction
**pAdCR5-GFP**	Activator/repressor	CR5	GFP	Transfer vector for generating adenovirus for screening cell lines stably expressing cTA.

#### Repressor configuration

##### pAdCMV5-CuOg-LacZ

A single operator site (CuO) has been introduced at the start site of CMV5, a strong constitutive promoter, driving the expression of the β-galactosidase reporter gene. The following steps were used to achieve this: A unique AgeI site was introduced in the promoter region of the CMV minimal promoter in pAdCR5-LacZ (described below) such that the site was 10 bp downstream of the TATA box using a PCR-based approach. Then, a 469 bp fragment corresponding to the promoter-enhancer region of CMV5 (-53 to -522) was amplified by PCR using pAdCMV5-K7-BFP [41] as the template. pAdCR5-LacZ-AgeI was digested with HindIII to remove the operator elements (6X-CuO) and the CMV5 PCR fragment was cloned as a HindIII fragment to generate pAdCMV5LacZ-AgeI. Complementary oligonucleotides were designed such that the ends of the annealed molecule were compatible with sticky end ligation in an AgeI-digested vector. The oligonucleotide contained one copy of the cumate operator sequence from P2 (P2-CuO: AACAAACAGACAATCTGGTC TGTTTGTA; Fig. 1A). The double stranded molecule was cloned into AgeI site of pAdCMV5-Og-LacZ. The AgeI site is 9 bp downstream of the TATA box.

##### pAdCMV5-CuOs-LacZ

pAdCR5-LacZ was digested with HindIII to remove the 6X-CuO and the 469 bp CMV5 promoter-enhancer PCR fragment (described above) was cloned into the HindIII site to give rise to pAdCMV5-LacZ. Complementary oligonucleotides were designed such that the ends of the annealed molecule were compatible with sticky end ligation in an AscI-digested vector. The oligonucleotide contained one copy of the cumate operator sequence from P2 (P2-CuO). The AscI site is at the start site of transcription.

##### pAdCMV5-CuO-GFP

pAdCMV5-GFP [29] was partially digested with BamHI and completely with XhoI. A 1084 bp fragment including the GFP coding sequence and a part of the 5' UTR sequences was cloned into pAdCMV5-CuOs-LacZ that was digested with XhoI (partially) and BamHI to remove the LacZ coding sequences. Thus pAdCMV5-CuO-GFP and pAdCMV5-CuOs-LacZ both contain the same promoter elements, but differ in the reporter gene (GFP and LacZ respectively) downstream of the promoter.

##### pAdCMV5-CymR-K7-BFP

pAdCMV5-CymR-K7-BFP is a plasmid with two expression cassettes, one expressing the repressor (CymR) under the control of the CMV5 promoter and the other, expressing BFP driven by the CMV promoter). The VP16 activation domain was excised from pAdCMV5-cTA-K7-BFP (described below), which is a similar vector expressing the chimaeric transactivator, CymR-VP16 or cTA. An intra-molecular ligation was carried out to generate pAdCMV5-CymR-K7-BFP. This plasmid will be referred to as pAdCMV5-CymR in the manuscript for the sake of brevity.

##### pMPG-BFP/CMV5-CymR/tk-neo

This expression plasmid for CymR (pMPG-CymR/tk-neo) was used to generate cell lines stably expressing the repressor. It comprises of three independent expression cassettes, a) one for the expression of CymR, using the CMV5 promoter, b) a second cassette where the CMV promoter drives BFP expression. BFP was used as a reporter gene to facilitate identification of transfected cells, and a third cassette for the expression of the G418 (neo)-resistance protein. It was derived from the pMPG series of vectors described in Gervais *et al*.[42]. pKCMVB43 [43] was digested with HindIII, rendered blunt and dephosphorylated and used to sub-clone the CymR coding sequence as a blunt-ended PCR fragment. The resulting plasmid was called pKCMV5-CymR. The CymR expression cassette (including the CMV5 promoter, the CymR coding sequence and the polyadenylation sequence) was excised as an AscI fragment from pKCMV5-CymR. This was then cloned into the AscI site of pMPG-BFP-tk-neo to give rise to pMPG-BFP/CMV5-CymR/tk-neo, referred to as pMPG-CymR/tk-neo in the manuscript for the sake of brevity.

#### Activator configuration

##### pAdCR5-LacZ

pAdCR5-LacZ was generated by removing the Tet operator sequences from pAdTR5-LacZ [41] and replacing them with the Cumate operator sequences. Therefore, pAdTR5-LacZ was digested with XhoI to remove the Tet operator sequences, the minimal promoter element and most of the Ad-tpl. The minimal promoter element and the Ad tpl were re-cloned into an XhoI-digested pAdTR5LacZ as a PCR fragment. The primers were designed such that the resulting fragment was flanked by XhoI sites and a new HindIII site was inserted. This intermediate vector was called pAdHindIIILacZ. A double stranded oligonucleotide (6X-CuO) containing six repetitions of the operator sequence from P1 (P1-CUO: AAAGAAACAAACCAACCTGTCTGT ATTATC) was then cloned into the HindIII site of pAdHindIIILacZ to give rise to pAd CR5-LacZ.

##### pAdCMV5-cTA-K7-BFP

pAdCMV5-cTA-K7-BFP is a plasmid with two expression cassettes, one expressing the activator (cTA) under the control of the CMV5 promoter and the other, expressing BFP driven by the CMV promoter). To generate the chimaeric transactivator, oligonucleotides were designed to perform a PCR reaction on the CymR coding sequence such that the initiator methionine was in the context of a Kozak sequence. Similarly oligonucleotides were designed to perform a PCR reaction on amino acid 363 to 490 of the herpes simplex virus protein 16 (VP16). The two PCR fragments were cloned into pAdCMV5-K7-BFP [41] in a three-way ligation to create an expression vector wherein the CMV5 promoter was driving the expression of the chimaeric protein, CymR-VP16 (pAdCMV5-cTA-K7BFP). This plasmid will be referred to as pAdCMV5-cTA in the manuscript for the sake of brevity.

##### pcDNA-cTA

A PCR fragment encompassing the cTA coding sequence was cloned in the multiple cloning site downstream of the CMV promoter in pcDNA-SEAP [44].

##### pAdPS-CMV-cTA-DC-GFP

To construct the transfer vector for generating the adenoviral library, pAdPS-CMV5-CuO-cTA-DC-GFP, a parental vector that expressed the adenoviral protease coding sequence (PS) under the control of the adenoviral major late promoter (mlp) [31], was digested with BglII to remove the CMV5-CuO promoter. Two PCR gragments, a) CMV promoter enhancer and b) cTA were cloned into the BglII-digested vector. This plasmid will be referred to as pAdPSCMV-cTA in this manuscript for the sake of brevity.

##### pRRL.cppt.CMV5-CuO-rcTA.WPRE

A SpeI-SmaI fragment encompassing part of the CMV5 promoter and the cTA coding sequence in pRRL.cppt.CMV5-cTA-WPRE (Broussau *et al *in preparation) was replaced by a SpeI-SmaI fragment (CMV5-CuO-rcTA) to give pRRL.cppt.CMV5-CuO-rcTA.WPRE.

##### pAdCR5-GFP

pAdCR5-GFP was generated from pAdTR5-GFP [41] by exchanging the Tet-regulated promoter for the cumate-regulated promoter (AflII-BlpI fragment).

##### pRRL.cppt.CR5-GFP.WPRE

The lentiviral backbone, pRRL.cppt.CR5-GFP.WPRE was derived from pRRL.cppt.hPGK-eGFP.WPRE [45] employing the following steps. A fragment of DNA encompassing the CMV5 promoter linked to the GFP coding sequences was amplified by PCR from the plasmid pUC19-CMV5-GFP. The DNA fragment was digested with ClaI, followed by treatment with T4 DNA polymerase to render the ClaI site blunt. This was then followed by a digestion with SalI. The hPGK promoter and eGFP sequences in pRRL.cppt.hPGK-eGFP.WPRE were replaced by the CMV5 promoter and GFP coding sequences respectively by excision of the former by digestion of pRRL.cppt.hPGK-eGFP.WPRE with EcoRV and SalI and followed by ligation with the CMV5-GFP fragment. The CMV5 promoter in pRRL.cppt.CMV5-GFP.WPRE was then replaced by the CR5 promoter from pAdPS-CR5mcs-IRES-GFP by exchanging the XhoI fragments of the two plasmids.

##### pRRL.cppt.CR5-SEAP-IRES-GFP

The AscI-SpeI (the AscI site digests in the CR5 sequence and SpeI in the GFP coding region) of pRRL.cppt.CR5-GFP.WPRE was replaced with that from pAdCR5-SEAP-IRES-GFP.

### Cells and transient transfection

293, A549, BMAdE1-78-42 (an A549 clone expressing the E1 region of Ad5) [46], and HeLa cells were maintained in DMEM supplemented with 10% heat inactivated FBS and 2 mM glutamine. All reagents for cell culture were purchased from Gibco-Invitrogen, (Burlington, ON, Canada).

Transfection conditions were optimized for each of the cell lines studies. 293 and HeLa cells were transfected using the calcium phosphate technique. One ml of DNA-calcium phosphate precipitate contained a total of 10 μg DNA (5 μg of reporter (pAdCMV5-CuOs-LacZ or pAdCMV5-CuOg-LacZ or pAdCR5-LacZ), 0.5 μg of repressor (pAdCMV5-CymR) or activator (pAdCMV5-cTA) and 3 μg of pcDNA-SEAP). This was divided equally between two 60 mm plates, each containing 10^6 ^293 cells. One of the two plates received in addition 200 μg/ml cumate. pcDNA-SEAP [44] was included in all transfections, so that secreted alkaline phosphatase (SEAP) activity could be used to normalize for transfection efficiency. 293CR5-LacZ were transfected with 400 ng of activator (pAdCMV5rcTA or pAdCMV5cTA) plasmid DNA and 2 μg of carrier DNA mixed with 4.8 μl of PEI in the presence and absence of 200 μg/ml of cumate. Transfections in A549 and BMAdE1-78 cells were carried out using Geneporter (Gene Therapy Systems Inc. San Diego, CA) according to manufacturer's directions. Briefly, 3 μg DNA (2 *μ*g pAdCR5-LacZ, 25 ng pAdCMV5-cTA and 0.5 *μ*g pcDNA-SEAP) in 500 μl DMEM was added to 1 × 10^5 ^A549 or 6 × 10^5 ^BMAdE1-78 cells in 500 μl DMEM. After 3 h, 1 ml DMEM was added to the plates so that the final concentration of serum was 10% and half the plates received cumate at a concentration of 200 μg/ml.

SEAP and β-galactosidase activity: SEAP activity was measured as described [44]. Briefly, 50 μl of 2X SEAP buffer (1 M diethanolamine pH 9.8, 2 mM MgCl_2_, 10 mM 1-homoarginine and 20 mM p-nitrophenyl phosphate, disodium, hexahydrate Sigma 104 phosphatase substrate; Sigma-Aldrich, Oakville, Ontario, Canada) was added to 50 μl of cell culture medium. OD_405 _was read using a plate reader after incubation at room temperature for different intervals of time. This information was used to ensure that the enzyme activity was measured under conditions where the substrate was in excess. Colorimetric assay for β-galactosidase (β-gal) activity was measured in transfected/infected cell extracts. Cells were lysed 48 h post-transfection by three freeze-thaw cycles in 0.25 M Tris.HCl pH 8. The cell lysate was centrifuged at 14,000 × g and enzyme (β-galactosidase) activity was measured in the supernatant (cell extract) using a colorimetric assay containing 1 mM MgCl2, 0.9 mg/ml O-nitrophenyl β-D-Galacto-pyranoside (ONPG), and 66 mM sodium phosphate (pH 7.5). Reactions were incubated at 37°C until a faint yellow color had developed. OD_420 _was measured at regular intervals until the reaction appeared to plateau. To stain transfected cells for β-galactosidase activity, cells were washed with PBS, fixed with glutaraldehyde (0.5% glutaraldehyde in PBS for 3 mins. at room temperature), washed again and incubated in β-galactosidase buffer (50 ml contains 1 ml of a 20 mg/ml X-gal solution in dimethylformamide, 0.05 gm magnesium chloride (1 mM), 0.082 gm potassium ferricyanide (5 mM) and 0.11 gm potassium ferrocyanide (5 mM)) for 4–16 h at room temperature in the dark at which point the β-galactosidase buffer was washed off [47].

### Stable cell lines

***293-CymR ***were generated by transfecting 1 × 10^6 ^293 cells with 10 μg of pMPG-CymR/tk-neo by the calcium phosphate technique. G418 (400 μg/ml) was added to the growth medium 48 h after the transfection to select a pool of G418-resistant cells. Individual clones (293-CymR) were isolated from this pool by the method of limiting dilution and tested for CymR function by infection with AdCMV5-CuO-LacZ.

***293CymR-CMV5-CuO-GFP ***clones were generated by transfecting 1 × 10^6 ^293-CymR clone 21 with 10 *μ*g of pAdCMV5-CuO-GFP. GFP expression was induced by the addition of 200 μg/ml cumate and individual GFP positive cells were picked by Quixell™ [48]. Total GFP fluorescence was measured using an EPICS™XL-flow cytofluorometer (Beckman Coulter, Fullerton, CA) equipped with 15 mW at 488 nm argon ion laser as an excitation source. The green fluorescence emission was detected using a 550 nm dichroic long pass and a 525 nm band pass filter set. The fluorescence index was calculated by multiplying the percentage of positive cells by the mean fluorescence value.

***293-cTA ***cells were generated as described above for 293CymR except that pcDNA3-cTA was used for the transfection. G418-resistant clones were tested by infection with AdCR5-GFP. ***293-cTA-CR5-GFP ***cells were generated by transduction of 293cTA cells with lenti-CR5-GFP. Four cell pools thus generated were analyzed by flow cytometry to identify those that had 5–20% of GFP positive cells (to assure one copy of integrated lentivirus genome per cell). The selected pool was enriched for GFP+ cells by subsequent GFP positive cell sorting using a Coulter EPICS™ -ESP cell sorter (Beckman-Coulter, Fl) using the same filter set as described earlier. The final percentage of GFP+ cells in the pool was 74%.

***293-CR5-LacZ ***were generated by transfecting 1 × 10^6 ^293 cells with 4 *μ*g of pAdCR5LacZ-neo using 8 μl of PEI reagent. G418 (400 μg/ml) was added to the growth medium 48 h after the transfection to select a pool of G418-resistant cells. Individual clones were isolated from this pool by the method of limiting dilution and verified by infection with AdCMV-cTA (see below).

#### 293rcTA and 293CymR-rcTA

Cell lines stably expressing the rcTA, either under the control of a constitutive promoter (CMV5) or under an inducible promoter (CMV5-CuO) were generated by transducing the parental cells 293 and 293CymR with a lentiviral vector lenti-CMV5-CuO-rcTA. The 293rcTA and 293CymR-rcTA pools were sub-cloned by limiting dilution. ***293rcTA-CR5-SEAP-IRES-GFP ***and ***293CymR-rcTA-CR5-SEAP-IRES-GFP ***pools were generated by transducing 293rcTAclone #40 and 293CymR-rcTAclone #18 with lenti-CR5-SEAP-IRES-GFP. The transduction was repeated twice at 24 h intervals to maximize the number of cells transduced.

### Virus production and infection

Recombinant AdVs were generated by *in vivo *homologous recombination between overlapping sequences of linearized transfer vectors (pAdCR5-GFP, pAdCMV5-CuO-LacZ and pAdCMV5-cTA) and Ad5/Δ E1Δ E3 in 293 cells as described in Massie *et al*. [29]. An initial screen for recombinants plaques was performed by visual examination for GFP expression (AdCR5-GFP) or β-gal activity (AdCMV5-CuO-LacZ). Transgene expression was verified by western analysis and activity was confirmed by co-infecting 293CR5LacZ (AdCMV5-cTA).

Lentivirus production: We have established a stable 293SF cell line (adapted to serum-free culture) expressing both the cumate repressor (CymR) and the reverse tetracycline transactivator (rtTA) for tight regulation of the packaging elements (293SF-PacLV). We co-transfected all packaging plasmids in one shot with Rev and VSV-G being doubly regulated. With the best clones generated, we obtained titers equal or greater than what is generated by transient transfection of the packaging constructs (> 5X10E6 transducing units/ml) (Broussau *et al. *manuscript in preparation). 293SF-PacLV cells (clone #16–22) were seeded in two 6 well plates at 0.75 × 10^6 ^cells per well in complete H-SFM [Hybridoma-SFM (H-SFM, Invitrogen, Grand Island, NY) supplemented with 0.1% Pluronic F-68 and 1% fetal bovine serum (FBS)]. The next day, media was changed 2 hours prior to transfection. 3 μg of plasmid (pRRL.cppt.CR5-GFP.WPRE, pRRL.cppt.CR5-SEAP-IRES-GFP.WPRE, pRRL.cppt.CMV5-CuO-rcTA.WPRE) was diluted in 100 μl of complete H-SFM without FBS and mixed with 6 μg of PEI (preparation for one well). This DNA/PEI complex was incubated at room temperature for 15 min before being added to the cells. The cell media was changed 4 hours post-transfection with 2 ml per well of complete H-SFM supplemented with 50 μg/ml cumate and 1 μg/ml doxycycline to induce packaging elements for lentivirus production. The supernatant was harvested 48 h post-transfection, filtered at 0.45 μm and stored at -80°C.

### Viral infections

#### Adenoviral infection

Cells were infected with the relevant viruses at a multiplicity of infection (MOI) of 10. Forty-eight hours post-infection, reporter gene expression levels were measured. For vectors with GFP as a reporter, total GFP fluorescence was measured by an EPICS™XL-flow cytofluorometer (Beckman Coulter). The fluorescence index was calculated by multiplying the percentage of positive cells by the mean fluorescence value. For viruses with LacZ as a reporter, β-galactosidase activity was measured as described above.

#### Lentiviral infection

The day before transduction, cells were seeded in a 12 well plate at 0.14 × 10^6 ^cells per well. Dilutions were prepared with 4 different amounts of the previously produced lentivirus: 250 μl, 166 μl, 50 μl and 16.6 μl diluted in Dulbecco's Modified Eagle Medium (DMEM) supplemented with 5% FBS for a total transduction volume of 500 μl supplemented with 8 μg of polybrene. The virus/polybrene preparations were pre-incubated 30 min at 37°C before adding to the cells and transductions were carried out overnight at 37°C. The media was changed for fresh DMEM followed by additional 48 h incubation at 37°C.

### Mutagenesis of CymR

The PCR-based random mutagenesis of CymR was carried out using GeneMorph™ PCR Mutagenesis Kit (Stratagene, La Jolla, CA) according to the manufacturer's instructions. Briefly, the plasmid pAdCMV5cTA was used as a template for CymR mutations and the PCR was performed with the primers 5'-TCCACTTTGCCTTTCTCTCC (forward primer) and 5'-GTTTTTCGTACGCGC GCGGCTGTACG (reverse primer) under conditions which would lead to frequent misincorporation of nucleotides. A total of three groups with different degrees of nucleotide misincorporation ranging from 0–3 mismatches per kb to 7–13 mismatches/kb were generated. All three groups were digested with BglII and NotI and ligated to the corresponding restricted pAd-PS-CMVcTA-IRES-GFP to substitute the wild-type CymR for the mutagenized ones. In order to optimize the diversity of the CymR mutations, electrophoretic transformation with *E. coli *Dh 5α was carried out and maxi prepared DNA was ready for further use.

#### Generation of CymR mutant recombinant adenovirus libraries with positive selection

The adenovirus positive selection system has been described previously (30). 293A cells were plated in 100 mm dish one day before the infection with modified adenovirus Ad5- PS at a MOI of 10^-2^. 5 h later, 10 μg DNA of linearized pAd-PS-CMV-mut-cTA-IRES-GFP plasmid and 20 ul of PEI were added to the 100 mm dish of infected 293A cells to generate recombinant Ad5-PS-CMV-mut-cTA-IRES-GFP libraries. 3 days later, when 90% of the cell population was positive for GFP expression, cells were harvested and subjected to 3 freeze-thaw cycles to release the recombinant viruses. The measurement of virus titers was done by plaque assay (5).

### Recombinant adenovirus library screening

293A-CR5-LacZ were plated in 100 mm dishes (5 × 10^6 ^per dish) and infected with 100–200 p.f.u./dish. After 6 hr infection, cells were washed and 10 ml of 5% agarose was added to each plate. After the appearance of plaques, to determine β-gal activity, the plates were overlayed with 5 ml of 1% sea plaque agarose in DMEM supplemented with 0.06% blue-gal in the presence or absence of 200 μg/ml of inducer cumate. Plaques were screened for β-gal activity in the presence of cumate. Positive plaques were purified three times for further analysis.

## Authors' contributions

AM participated in the planning of experiments, data analysis and wrote the manuscript.

YX generated the reverse mutant.

RW generated the plasmids for the repressor configuration of the switch and carried out the transient transfections in 293 cells (Fig. 2A).

MK generated the 293-CymR clone 21 cell line (Fig. 2B and 2C)

CG generated the 293-cTA cell line and produced the adenoviral constructs

FM studied the performance of the switch (activator configuration) in 293, A549, BMAdE1-78 and HeLa cells (Fig. 3A)

LL generated the plasmids for the activator configuration of the switch.

LB performed and contributed to the interpretation of the flow cytometry experiments.

SB generated the lentiviral vectors and stable cell pools by lentiviral transduction.

RL and AP compared the activity and expression of the cTA and rcTA using transient transfections (Fig 6B).

AWC performed the cumate dose-response for 293CymR-CMV5-CuO-GFP and 293cTA-CR5-GFP (Fig 4)

BM The initial idea came from BM. He was involved in the planning and analysis of the experiments and the writing of the manuscript.

All authors have read and approved the final manuscript.
